# Revisiting relationships between developmental trauma and violence

**DOI:** 10.3389/fpsyt.2024.1401742

**Published:** 2024-07-30

**Authors:** Rithvic Jupudi, Katarina Bojkovic, Anthony Schinelli, Brooke Jennings, Robert Bossarte

**Affiliations:** ^1^ University of South Florida Health Morsani College of Medicine, Tampa, FL, United States; ^2^ Department of Psychiatry and Behavioral Neurosciences, University of South Florida, Tampa, FL, United States

**Keywords:** developmental trauma, adolescent psychiatry, violence, mental health, gun violence prevention

## Introduction: relationships between mental health and violence

Violent crime, perpetrated by persons of all ages, has garnered significant popular and scientific media attention. In the wake of tragedies, much discourse has focused on associations between mental illness and violence (1), particularly when firearms are involved. However, the exact relationships between mental illness and violence or, more importantly, proposed solutions to mental health needs among those at risk for perpetrating violence are less clear. Considering current social landscape, it is important to understand what is known about links between mental illness and violence. Many correlate the two, but the conversation suffers from the non-inclusion of trauma. Understanding this is needed to both develop evidence-based treatment options and prevent promulgation of misdirected narratives.

Within clinical and academic spheres, there exists a consensus that attributing violence to mental illness is an oversimplification of the multifaceted causative issues. Nevertheless, there is merit in a deeper discussion of the relationships between mental health and violence, and, most importantly, gaps in our current efforts to develop service models tailored to the needs of persons at risk, specifically for gun violence. We believe the first step in this process is demonstrating a relationship between childhood trauma and violence. We hope to reintroduce developmental trauma disorder (DTD), to illuminate how the developmental stage one is at when experiencing trauma has unique implications through the rest of one’s life. Due to both the scale of the problem and system-supported opportunities for delivery of services, we will limit our discussion to characteristics and services targeting those 18 years and younger.

## What do we know about the mental health of those at risk for violence?

We conducted a literature review using key terms: “violence, mental illness/health, juvenile guns, trauma, developmental trauma disorder, and risk factors.” We identified 52 PubMed indexed articles included in the final analysis, excluding studies over 15 years old and outside the USA. Papers were restricted to the USA due to cultural familiarity as well as relative uniqueness of youth gun violence. However, careful consideration was given to all acts of violence, and not gun violence alone. There were no restrictions regarding publication type, with the majority being retrospective cohort, review, and small-scale observation studies. The search was limited to the past 15 years because Van Der Kolk introduced DTD in 2005, and it was rejected by the DSM-V in 2011. We believe conversation around DTD to be more important than ever, and the timeframe highlights this.

Existing literature suggests that as much as 50-70% of youth involved in the juvenile justice system have a diagnosable mental health condition in contrast with the 10-20% of juveniles in the general population (2). In one study evaluating the prevalence of mental health conditions among first-time juvenile offenders, the investigators reported that 74.3% of individuals in their sample met criteria for Oppositional Defiant Disorder, Conduct Disorder, Attention Deficit Hyperactive Disorder, Depression, or Anxiety (2). Specifically, symptoms consistent with a diagnosis of Oppositional Defiant Disorder were present in 43% of the study population, Conduct Disorder 37%, Attention Deficit Hyperactive Disorder 13%, Depression 13%, Anxiety 29%, and 50% met the criteria for more than one disorder (2).

## The importance of understanding trauma and trauma-informed care

Early exposure to trauma and resulting stress is a particularly important risk factor for psychiatric disorders, violence, and other adverse outcomes. Previous research has suggested that as much as 90% of youth involved with the U.S. criminal justice system have histories of repeated and significant traumatic exposure (3). Recent focus on the consequences of adverse childhood experiences has increased awareness of developmental consequences of early and repeated adversity. For example, there is evidence of high rates of substance abuse among youth who are arrested (3). Burke et al. highlight further that probability of having a psychiatric diagnosis increases with involvement with the juvenile system. In the same study, incarcerated youth were only referred out for psychiatric treatment if they had ODD or family communication issues and were not referred for care if they cited having high mental health burden (2). This shows again the top-down approach that predominates, with more attention to behavioral manifestations. Accordingly, there is need for a holistic consideration of lived experiences among perpetrators/victims of violence and the development of trauma-informed models of care. We must also acknowledge that those who experience trauma as children are more likely to be further victims or perpetrators of violence as adults, confirming the necessity of interrupting the cycle (4). From a systemic standpoint, trauma is compounded by profound mistrust in institutions, resulting from complex histories of marginalization and limited access to resources. The resultant dearth of resources and funding for prolonged care makes addressing these traumas a considerable challenge. Treatment models must be scalable and readily available to larger populations.

## Developmental trauma disorder

In 2005, Van Der Kolk and colleagues proposed inclusion of “Developmental Trauma Disorder” (DTD) in the DSM-V, after observation that trauma experienced at different ages during childhood presents with more distinct and complex sequelae than adults or those with less extensive exposure to traumatic events (4). Developmental Trauma Disorder as a diagnosis was proposed to help understand “triggered dysregulation in response to traumatic reminders, stimulus generalization, and anticipatory organization of behavior to prevent the recurrence of the trauma effects” (4). This essentially refers to strong reactions and an inability to respond in a healthy manner to reminders of trauma or stimuli that would otherwise not affect an emotionally secure child. Fear, rage, and avoidance are common themes seen in those who meet criteria for DTD (4). In children, a lack of acknowledgement of chronic interpersonal trauma can lead to frequently missed diagnoses, wrong diagnoses, and excessive emphasis on behavior control in clinical settings, resulting in an inability to address and ameliorate developmental disruptions underlying the observed symptoms (4). Relatedly, the team observed that children who experienced repeated traumatic exposures experienced similar manifestations later in life. In one instance, children who grew up in environments where domestic violence and/or alcoholic behavior was present consistently exhibited self-destructive and impulsive behavior along with depression in adulthood (4). In the researchers’ opinion, a lone diagnosis of PTSD did not encompass the cumulative effect of trauma during the important period of neurobiological and behavioral change that occurs during childhood. The list of such effects of trauma can range from altered world views, loss of autonomy, and loss of bodily regulation/somatic issues among others (4).

In 2021, the APA recognized the continuing importance of deeper levels of understanding of childhood trauma. Developmental trauma may not present with characteristic symptoms of PTSD, potentially leading to a slew of misdiagnoses and medication regimens. Children may be diagnosed with ADHD or ODD when they do not apply or do not properly encompass the whole of their psychopathological state (5). The DTD discussion can help us understand how trauma manifests at different ages and the multiplicative effect these exposures can have on risk for future adversity. Dr. Julian Ford asserts that DTD makes children feel as if they have something wrong with them due to inability to regulate their emotions, a result of their childhood trauma and constant fight or flight during this important developmental period (6, pp.31–33). The neurobiological model helps explain the physiological reactions. The overactivation of the stress response system in traumatized children can lead to depressed immune activity, decreased tissue and organ healing, and increased autoimmune activity (6, pp.31–33). This all occurs while depressing the dopaminergic centers of the brain involved in learning, serotonin dependent centers managing distress, and executive functions such as judgement (6, pp.31–33). These lead to an imbalance between the “survival brain” and the “learning brain,” which is detrimental to development. Relatedly, in juvenile delinquency studies, there is neurobiological fMRI evidence that demonstrates there are limitations in recruiting brain areas that plan response, process errors, and facilitate top-down executive function during adolescence (7). These do not absolve youth of their decision making but may be used to inform how we must ultimately invest in rehabilitation such as comorbid psychiatric treatment.

## Complex PTSD, PTSD with comorbidities, or something meaningfully different?

Despite the novelty of the DTD discussion, we would be remiss to not mention the persisting debate regarding developmental trauma as a unique and identifiable disorder or complex form of PTSD (CPTSD). The distinction is not just academic and has important consequences for assessment and treatment. In practice, PTSD is rarely diagnosed without comorbidities.

## Applicability to the current practice of psychiatry

The CDC released a 6-prong approach to trauma informed care (safety, trustworthiness & transparency, collaboration & mutuality, peer support, empowerment voice & choice, and cultural, historical, & gender issues) (8). Trauma informed care is the forefront of more pointed interventions. Extending this to developmental trauma informed care may be the logical next avenue. In the UK, a trauma informed intervention program, RedThread, centers on reaching youth (11-24) involved in violence who presented to the emergency department (9). They found youth opened up to the workers. Subsequently, information about patients found by RedThread positively impacted clinical decisions physicians made and underscored a clinically significant need for trauma informed care and stronger links between community and doctor. Peer support models and digital mental health care are potential treatment practices to reach large scale. Peer support has deep rooted history within many paradigms such as substance abuse, showing significant improvements in self efficacy, for one example (6, pp.31–33). Within communities suffering from violence experiencing mental health concerns, peer support may be a way of helping those who “may have felt something was wrong with them,” as Dr. Ford describes, re-assume control in their lives by being a part of the solution (6, pp.31–33). This model may curtail noncompliance, a regular issue with mental health treatment. Involving peer supporters from the same community as those being treated may strengthen the relationship between patients and the healthcare system and re-establish credibility. Further, digital mental health intervention (DHMI) can be a way of reaching underinsured populations. This solution will need improved cultural competency, but it offers an opportunity to combine peer support with a lower cost evidence based mental health treatment.

## Discussion

There is ample evidence that children considered to be at risk for violence have likely experienced significant trauma manifesting in a complex sequala of behaviors and negative life circumstances. We must take a deep look at our current toolbox for treating youth with complex PTSD and reflect on whether current frameworks for trauma informed care can extend to developmental trauma. This may be an opportunity to employ new, pointed interventions as a behavioral health community, or also an opportunity to simply get the diagnosis right in the first place. Regardless, solutions must be scalable and cost effective for low resource persons in low resource settings. This literature review was USA based but these problems and solutions are applicable worldwide. The framework for trauma informed care is universal, as demonstrated by programs such as RedThread in the UK. Long term outcome measurement should focus on rates of violence amongst youth with correctly identified psychiatric illnesses, repeat involvement in the juvenile justice system, and quality of life.

We call on psychiatry clinicians to help us understand children who have experienced complex and repeated trauma. This is an opportunity to practice precision psychiatry where we align treatment strategies with individual experience, rather than trying to fit unique manifestations into boxes. The current discussion surrounding the relationship between DTD and PTSD among the psychiatry/psychology community notwithstanding, we call for action to increase the level of attention given by clinicians towards the impact of trauma during critical developmental stages.

## Author contributions

RJ: Writing – review & editing, Writing – original draft, Methodology, Investigation, Formal analysis, Conceptualization. KB: Writing – review & editing, Validation, Methodology, Formal analysis, Conceptualization. AS: Writing – review & editing, Validation, Methodology, Formal analysis, Conceptualization. BJ: Writing – review & editing, Resources, Project administration, Formal analysis, Data curation. RB: Writing – review & editing, Writing – original draft, Validation, Supervision, Resources, Project administration, Methodology, Investigation, Formal analysis, Conceptualization.
